# Longitudinal study of knee pain amongst workers in the Cultural and Psychosocial Influences on Disability (CUPID) study

**DOI:** 10.1186/s12891-025-09237-z

**Published:** 2025-11-05

**Authors:** G. Ntani, V. E. Felli, F. Harari, L. H. Barrero, M. Rojas, C. Serra, M. Bonzini, E. Merisalu, R. R. Habib, F. Sadeghian, A. R. Wickremasinghe, K. Matsudaira, H. L. Kelsall, H. Harcombe, K. Walker-Bone

**Affiliations:** 1 Medical Research Council Lifecourse Epidemiology Centre, University of Southampton, Southampton, UK; 2 Medical Research Council Versus Arthritis Centre for Musculoskeletal Health and Work, Medical Research Council Lifecourse Epidemiology Unit, University of Southampton, Southampton, UK; 3 School of Nursing, University of São Paulo, São Paulo, Brazil; 4 Corporación para el Desarrollo de la Producción y el Medio Ambiente Laboral – IFA (Institute for the Development of Production and the Work Environment), Quito, Ecuador; 5 Occupational and Environmental Medicine, Sahlgrenska University Hospital and University of Gothenburg, Gothenburg, Sweden; 6 Department of Industrial Engineering, School of Engineering, Pontificia Universidad Javeriana, Bogotá, Colombia; 7 Program Health, Work and Environment in Central America, Institute for Studies On Toxic Substances (IRET), National University of Costa Rica, Heredia, Costa Rica; 8 Center for Research in Occupational Health (CiSAL), IMIM (Hospital del Mar Medical Research Institute)/University Pompeu Fabra, Barcelona, Spain; 9 CIBER of Epidemiology and Public Health, Madrid, Spain; 10 Occupational Health Service, Hospital del MAR, Barcelona, Spain; 11 University of Milan and IRCCS Ospedale Maggiore Policlinico Foudation, Milan, Italy; 12 Institute of Forestry and Engineering, Estonian University of Life Sciences, Tartu, Estonia; 13 Department of Environmental Health, Faculty of Health Sciences, American University of Beirut, Beirut, Lebanon; 14 Center for Health Related Social and Behavioral Sciences Research, Shahroud University of Medical Sciences, Shahroud, Iran; 15 Department of Public Health, Faculty of Medicine, University of Kelaniya, Ragama, Sri Lanka; 16 Department of Pain Medicine, Fukushima Medical University School of Medicine, Fukushima, Japan; 17 Monash Centre for Occupational and Environmental Health, School of Public Health and Preventive Medicine, Monash University, Melbourne, VIC Australia; 18 Department of Preventive and Social Medicine, University of Otago, Dunedin, New Zealand; 19 Monash Centre for Occupational and Environmental Health, Monash University, Melbourne, Australia

**Keywords:** Knee pain, Multisite pain, Work disability, Psychosocial, Occupational physical demands, Central sensitisation

## Abstract

**Background:**

Knee pain is common in the general population, increasingly so with age. It causes substantial disability which can lead to premature exit from the workforce. Current epidemiological data on knee pain prevalence in working-age populations are limited, particularly concerning the interplay of occupational, psychosocial, and cultural factors. To address this, in a large group of workers, we examined the prevalence of knee pain longitudinally, its relationship with pain at other sites and personal and occupational risk factors for pain at follow-up.

**Methods:**

Data came from the CUPID study, a cohort study including people aged 20–59 years at work from 18 countries in broadly three types of occupations: office work, nurses and manual workers. Baseline data were collected on knee pain, pain at other anatomical sites, occupational characteristics and psychosocial aspects of work. Follow-up information about knee pain was obtained approximately a year later. Descriptive statistics were used to describe knee pain prevalence and characteristics as well as persistence. Poisson regression was used to explore baseline factors in relation to knee pain, and disabling knee pain, at follow-up.

**Results:**

In total 9,161 workers provided baseline and follow-up data, amongst whom 28% reported knee pain in the past year and 21% knee pain in the past month at baseline. 79% of workers with knee pain reported disability. The prevalence increased with age and was higher among women. Only 8% of workers with knee pain had single-site pain. There was wide variation in the prevalence rates reported amongst workers even doing broadly similar work. Psychosocial and occupational work demands predicted knee pain at follow-up, as did poorer mental health and somatisation but the highest effect size was found amongst people reporting a high number of painful sites at baseline (PRR: 2.06,95%CI: 1.78–2.39).

**Conclusions:**

Knee pain is prevalent in the workforce, even at younger ages. It is commonly persistent and disabling. Given its link with work disability, the emphasis needs to be on prevention and both mechanical and psychosocial exposures at work are implicated. However, like low back pain, other factors appear to be important, particularly pain at other sites.

**Trial registration:**

Clinical trial number: not applicable.

**Supplementary Information:**

The online version contains supplementary material available at 10.1186/s12891-025-09237-z.

## Introduction

Knee pain is common in the general population. To illustrate, approximately a quarter of an English cohort of men and women aged > 55 years reported that they ever had knee pain lasting at least a month [1]. Similarly, as many as 25% of people in the same age range reported knee pain on most days for a month in the past year [2]. While osteoarthritis (OA) is the most common cause of knee pain, and knee cartilage injury is a significant cause among younger workers, especially men [3, 4], there are several other known causes [5]. Incidence of OA increases with age and consequently, a lot of the research focus on knee pain has been amongst the elderly [6]. However, there is a notable lack of robust epidemiological data on the prevalence of knee pain in people at work, limiting our understanding of the burden of knee pain among this group. Furthermore, existing studies often fail to account for the interplay between occupational, psychosocial, and cultural factors, which may differ significantly between older and younger adults.

Population studies in the Netherlands and Korea reported rates of knee pain of 22% [7], and 13.7% respectively [8]. Additionally, 13% of women aged 45–65 years reported this symptom [9]. With demographic shifts and rising obesity rates, it is anticipated that the incidence and prevalence of knee pain will increase [5]. Simultaneously, a growing proportion of the population is expected to continue working to older ages, potentially resulting in an increased prevalence of knee pain among the working population. However, knee pain can be disabling and can increase the risk of premature exit from paid work [10].

Recognised risk factors for knee pain include older age, obesity, female sex [11], and sedentary behaviour [12]. Occupational activities that have also been identified as risk factors for knee OA include heavy lifting and kneeling/squatting, particularly in combination [13]. However, knee pain tends to occur as part of a wider tendency to report musculoskeletal pain [14]. Indeed, amongst US adults aged 45–74 years, it was found that traditional risk factors for radiographic OA were not associated with knee pain, whereas factors known to affect wider propensity to pain, such as psychological wellbeing and self-reported health status, were more strongly associated with knee pain [15]. Despite these findings, many studies lack a longitudinal design or comprehensive assessment of work-related and psychosocial factors, resulting in fragmented evidence. Additionally, there is limited understanding of how factors such as culture, sex, or broader pain-processing mechanisms interact with occupational risks to exacerbate knee pain [16, 17]. In this respect, knee pain is perhaps similar to musculoskeletal pain at other body sites, in which it is determined by an interplay between structural damage, pain processing mechanisms, and culture, sex, and psychosocial factors [16, 17]. Recent studies of musculoskeletal pain have further demonstrated that self-reported disabling pain in the lower back and wrist/hand was strongly related to the extent of pain at other anatomical sites, assessed some 14 months earlier [18, 19]. Amongst people with or at risk of OA, it was found that individuals with bilateral and unilateral knee pain were more likely than people without knee pain to develop pain at other musculoskeletal sites during follow-up [15]. These limitations in the existing literature highlight the need for comprehensive studies that integrate both occupational and psychosocial dimensions of knee pain.

Given that knee pain can affect ability to work and can be caused by work factors, the aim of this study was to describe the prevalence and characteristics of knee pain over 12 months of follow-up in a group of workers aged between 20 and 59 recruited from 18 countries. Additionally, we aimed to investigate the effects of personal risk factors, psychosocial aspects of work, occupational physical demands, and the relative effect of the number of anatomical sites reported as painful in the month before baseline, previously referred to as propensity of musculoskeletal pain [18].

## Methods

The CUPID study, detailed elsewhere [20], is an international cohort of participants aged 20–59 years at baseline, employed in their current job for at least 12 months, recruited between 2006–2011. Participants were recruited from 18 countries, where local investigators recruited samples of nurses, office workers who regularly used computer keyboards and/or mice, and workers performing repetitive manual tasks with their arms or hands (detailed description of each group can be found in Table 1 of [20]). The final sample consisted of 47 occupational groups (group of workers by country), mainly nurses (42%), office workers (31%), and manual workers (27%). Participants, employed for at least 12 months, completed a baseline questionnaire with a 70% response rate. The questionnaire, drafted in English and then independently back-translated for accuracy, gathered data on demographics, lifestyle, work characteristics, occupational physical demands (does an average day at work involve any of the following activities? kneeling/squatting for > 1 h/day; lifting weights ≥ 25 kg; climbing stairs > 30 flights of stairs per day – response options yes/no), psychosocial factors (working > 50 h/week time pressure at work; workplace demands; control over work; support at work; job security) mental health, and somatising tendency. Mental health was assessed via the Short Form-36 grouped into three-levels (‘good’, ‘intermediate’, and ‘poor’) representing approximate thirds of the distribution of scores in the full study sample. Somatisation was assessed through the Brief Symptom Inventory and was categorised according to the number of common somatic symptoms experienced from a total of five.

In the baseline questionnaire, participants were asked if they had experienced pain in the past month or past 12 months in 10 anatomical areas (low back, neck, and both shoulders, elbows, wrists/hands, and knees) using body diagrams. Questions covered pain duration, related disability (pain in the past month), need for medical consultation, and any related sickness absence. Localised knee pain at baseline was defined as knee pain in the past month without pain in other areas during the previous 12 months, while non-localised knee pain was defined as knee pain with additional pain in other areas during the same period.

Approximately one year after the baseline, participants filled out a follow-up questionnaire. This time, they were again asked about any pain lasting a day or more in the past month in each of the 10 anatomical areas. Disabling knee pain (at either baseline or follow-up) was defined as knee pain that made it difficult or impossible to perform any of four specific activities: climbing stairs, walking on flat ground, getting dressed, or doing regular household tasks.

Information of the data gathered and the specific questions used in the questionnaire developed for this study can be found in the original methods paper [20].

### Statistical analysis

Baseline characteristics of knee pain in the past 12 months before baseline were summarised by prevalence rates and 95% confidence intervals. Incidence and persistence of knee pain over one year of follow-up for each of the 45 occupational groups were described graphically. We then applied Poisson regression to explore associations of knee pain and disabling knee pain in the past month before follow-up with risk factors assessed at baseline. Risk estimates were summarised by prevalence rate ratios (PRRs) with robust standard errors, and to account for possible clustering by occupational group, we applied random intercept modelling. The risk factors explored were sociodemographic characteristics, occupational activities (lifting weights ≥ 25 kg, climbing up or down more than 30 flights of stairs, and kneeling or squatting for longer than an hour on an average working day), psychosocial aspects of work, mental health, somatisation, and number of anatomical sites with pain in the past 12 months before baseline (excluding pain in the knee and lower back). Each of those risk factors was initially minimally adjusted (for sex and age only), and then those significant at the 5% level from the minimally adjusted models were included in a mutually adjusted model with and without further adjustment for the number of painful anatomical sites in the past 12 months before baseline.

All analyses were carried out using Stata v.12.1 software (Stata Corp LP 2012, Stata Statistical Software: Release 12.1, College Station TX, USA).

## Results

From the original 47 occupational groups, 45 were successfully followed up approximately 14 months after baseline (only manual workers in Costa Rica and office workers in South Africa were not). In these groups, a total of 11,992 returned a baseline questionnaire, of which 11,835 provided adequate information about pain at different anatomical sites in the 12 months before baseline. Of these, 9,161 (77%) returned a follow-up questionnaire with information about knee pain in the past month. Occupational groups varied in sizes from 42 manual workers in Brazil to 656 manual workers in Japan (further details on sample sizes and response rates by occupation groups are provided in Supplementary Table A1). Response rates ranged from 39 to 97% across the 45 occupational groups, and were somewhat higher among office workers, non-smokers, those with good mental health, and those with knee pain at baseline (Table 1).

**Table 1:** Characteristics of participants in the CUPID study at baseline and response rates at follow-up

Baseline characteristics	Number with baseline data	Number with follow-up data	Response rates (%)
Sex			
Male	4,119	3,132	76.0%
Female	7,716	6,029	78.1%
Age (years)			
20–29	2,838	2,115	74.5%
30–39	3,813	2,933	76.9%
40–49	3,327	2,634	79.2%
50–59	1,857	1,479	79.6%
Occupation			
Nurse	5,031	3,820	75.9%
Office worker	3,458	2,879	83.3%
Other worker	3,346	2,462	73.6%
Smoking			
Never	7,459	5,900	79.1%
Former smoker	1,707	1,311	76.8%
Current smoker	2,630	1,926	73.2%
Unknown	39	24	61.5%
Mental Health			
Good	4,492	3,623	80.7%
Intermediate	3,597	2,771	77.0%
Poor	3,677	2,733	74.3%
Unknown	69	34	49.3%
Number of distressing somatic symptoms			
0	7,188	5,488	76.4%
1	2,487	1,991	80.1%
2 +	2,055	1,628	79.2%
Unknown	105	54	51.4%
Knee pain in past 12 months			
No	9,456	7,263	76.8%
Yes	2,379	1,898	79.8%
Knee pain in the past month			
No	8,561	6,578	76.8%
Yes	3,274	2,583	78.9%
All participants	11,835	9,161	77.4%

At baseline, 2,583 (28.2%) participants reported knee pain in the past 12 months, 1,898 (73.5%) of whom reported having knee pain during the past month. One-year prevalence of knee pain was higher in females compared with males, and increased with age (20–29 years: 22.0%; 30–39 years: 24.8%; 40–49 years: 31.5%; 50–59 years: 38.0%). Patterns for sex and age were similar for one-month prevalence of knee pain.

Baseline characteristics of participants reporting knee pain in the past 12 months at baseline are shown in Table 2. Of those reporting knee pain the past 12 months, most (73%) reported pain in the past month, of whom half reported that their pain lasted over a week. Of those reporting knee pain in the past 12 months at baseline, approximately one-third reported that it lasted for longer than a month. The vast majority of people reporting knee pain in the past month at baseline also reported disability from the pain (79%), while 41% of the knee pain cases in the past 12 months before baseline sought medical care, and 15% went off sick for at least one day due to their pain. Over 90% of people reporting knee pain reported pain in at least one other anatomical site.

**Table 2:** Baseline characteristics of participants reporting knee pain in the past 12 months at baseline (reported by *N* = 2,583)

	N	%	(95%CI)
Sex			
Male	722	28.0	(26.2–29.7)
Female	1,861	72.0	(70.3–73.8)
Age (years)			
20–29	465	18.0	(16.5–19.5)
30–39	727	28.1	(26.4–29.9)
40–49	829	32.1	(30.3–33.9)
50–59	562	21.8	(20.2–23.4)
Total duration in the past month			
0 days	685	26.5	(24.8–28.3)
1–6 days	926	35.9	(34.0–37.7)
1–2 weeks	392	15.2	(13.8–16.6)
> 2 weeks	568	22.0	(20.4–23.6)
Not known	12	0.5	
Total duration in the past 12 months			
1–6 days	872	33.8	(31.9–35.6)
1–4 weeks	954	36.9	(35.1–38.8)
1–12 months	739	28.6	(26.9–30.4)
Not known	18	0.7	
Disabling in past month^1^	1,504	79.2	(77.3–81.0)
Led to medical consultation in past 12 months	1,050	40.7	(38.7–42.6)
Attributed sickness absence in past 12 months (days)			
0	2,198	85.1	(83.7–86.4)
1–5	236	9.1	(8.1–10.3)
6–30	84	3.3	(2.6–4.0)
> 30	56	2.2	(1.6–2.8)
Not known	9	0.4	
Localised			
No	2,365	91.6	(90.4–92.6)
Yes	218	8.4	(7.4–9.6)

^1^Calculated among 1,898 with knee pain in the past month before baseline

**Fig. 1:**
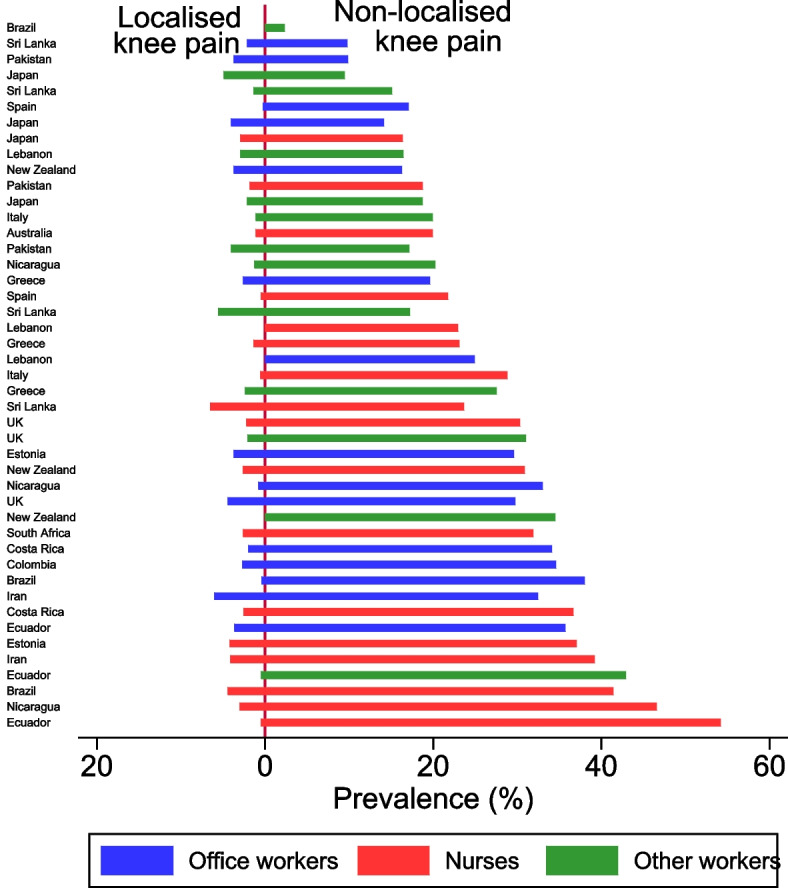
12-month prevalence of non-localised and localised knee pain reported at baseline by occupational group (ranked by overall one-year prevalence of knee pain at baseline)

The prevalence of knee pain in the past 12 months at baseline varied widely across the 45 occupational groups in the study. Figure 1 shows that the prevalence of knee pain only (to the left of the y axis) is much lower than the prevalence of knee pain reported with pain in at least one other anatomical site (to the right of the y axis) in all 47 occupational groups. Moreover, the prevalence in any one occupational group e.g. manual workers varies enormously in people doing the same type of work but in a different country (< 3% of manual workers in Brazil as compared with 44% of manual workers in Ecuador). Additionally, the prevalence of knee pain appears to be more similar within countries amongst people doing different types of work e.g. Japanese office workers, Japanese nurse and Japanese manual workers as compared with workers form all three groups in Ecuador).

**Fig. 2:**
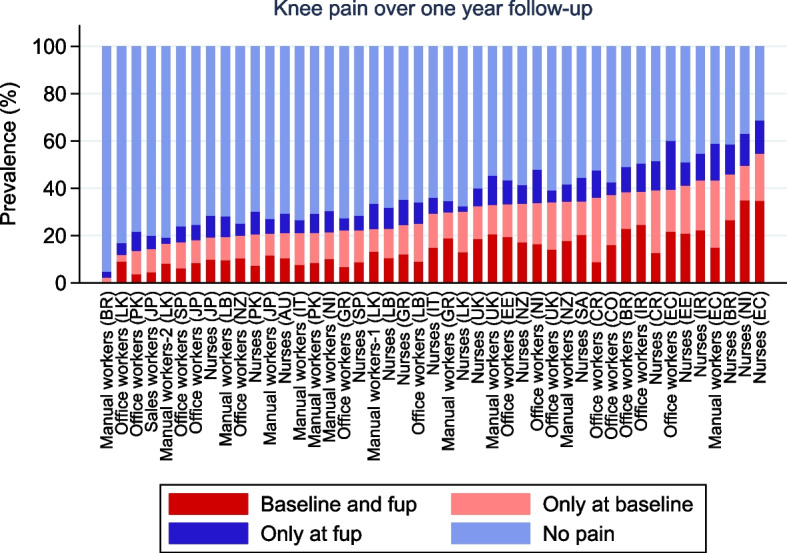
Knee pain over one year of follow-up

Figure 2 shows the variation in prevalence of knee pain over one year of follow-up in the different occupational groups. Overall, approximately half of participants who reported knee pain in the past 12 months at baseline also reported knee pain at follow-up (i.e. persistent pain), while among those without knee pain at baseline only 12.2% reported pain at follow-up (incident pain). Both rates of persistent and incident knee pain ranged considerably across occupational groups (from 0%−76.5%, and from 2.4%−34.3% respectively).

Table 3 summarises the associations of knee pain reported at follow-up with personal and occupational risk factors assessed at baseline, initially minimally adjusted (for sex and age only) and subsequently mutually adjusted with and without adjustment also for number of anatomical sites with pain in the past 12 months at baseline. In the minimally adjusted models, we found that risk of knee pain was notably higher among those in the age group 30–39 as compared with those aged 20–29 (PRR: 1.19, 95% CI: 1.03, 1.36), with the risk increasing further with each higher age. Occupational physical demands also showed an elevated risk of knee pain at follow-up (PRR: 1.2 for both climbing up/down stairs, and kneeling or squatting), as did several psychosocial aspects of work, including time pressure (PRR: 1.26, 95% CI: 1.13, 1.41), lack of support (PRR: 1.18, 95% CI: 1.08, 1.29) and job insecurity (PRR: 1.1, 95% CI: 1.02, 1.20). Higher risks were estimated for participants with greater tendency to somatise (PRR: 1.54, 95% CI: 1.39, 1.71, for report of ≥ 2 vs. 0 distressing somatic symptoms), poor as compared with good mental health (PRR: 1.37, 95% CI: 1.24, 1.51), and for increasing number of painful sites at baseline (PRRs increasing up to 2.56 for 4 + painful sites vs no pain at baseline). Except for the effect of job insecurity, all the other sex- and age-adjusted effects significantly associated with knee pain were only slightly attenuated after mutual adjustment, with and without extra adjustment for number of sites with pain at baseline. However, after allowance for all covariates, number of painful anatomical sites reported at baseline was the strongest risk factor for knee pain at follow-up. When analyses were repeated using disabling knee pain at follow-up as an outcome, associations were very similar (Supplementary Table A2).

**Table 3:** Associations of knee pain in the past month as reported at follow-up with personal and occupational risk factors assessed at baseline

Risk factor	All	Knee pain at follow-up	Sex- and age-adjusted estimates		Mutually adjusted estimates^2^		Mutually adjusted estimates^2^	
	**N**	**N (%)**	**PRR**	**(95%CI)**	**PRR**	**(95%CI)**	**PRR**	**(95%CI)**
Sex								
Male	3,132	572 (18.3)	1		1		1	
Female	6,029	1,510 (25.0)	1.18	(1.04,1.34)	1.1	(0.97,1.25)	1.04	(0.91,1.17)
Age (years)								
20–29	2,115	355 (16.8)	1		1		1	
30–39	2,933	590 (20.1)	1.19	(1.03,1.36)	1.21	(1.06,1.38)	1.16	(1.02,1.32)
40–49	2,634	665 (25.2)	1.45	(1.27,1.64)	1.5	(1.33,1.68)	1.38	(1.23,1.55)
50–59	1,479	472 (31.9)	1.83	(1.60,2.10)	1.91	(1.68,2.16)	1.71	(1.51,1.92)
Smoking								
Never smoked	5,900	1,434 (24.3)	1		1		1	
Ex-smoker	1,311	292 (22.3)	0.98	(0.87,1.10)	0.97	(0.86,1.09)	0.95	(0.84,1.06)
Current smoker	1,926	349 (18.1)	0.89	(0.80,0.99)	0.84	(0.75,0.93)	0.82	(0.73,0.91)
Not known	24	7 (29.2)						
Activity in average working day								
Lifting weights ≥ 25 kg	3,279	760 (23.2)	1.09	(0.98,1.21)				
Climbing up or down more than 30 flights of stairs	2,068	552 (26.7)	1.21	(1.06,1.37)	1.16	(1.03,1.29)	1.16	(1.03,1.30)
Kneeling or squatting for > 1 h	2,469	654 (26.5)	1.22	(1.12,1.33)	1.15	(1.06,1.25)	1.1	(1.01,1.20)
Psychosocial aspects of work								
Work for > 50 h per week	2,069	390 (18.8)	1.03	(0.91,1.15)				
Time pressure at work	6,831	1,615 (23.6)	1.26	(1.13,1.41)	1.19	(1.07,1.32)	1.15	(1.04,1.29)
Incentives at work	2,528	578 (22.9)	1.07	(0.97,1.19)				
Lack of support at work	2,358	626 (26.5)	1.18	(1.08,1.29)	1.16	(1.06,1.26)	1.13	(1.04,1.23)
Job dissatisfaction	1,781	381 (21.4)	1.05	(0.94,1.17)				
Lack of job control	1,840	410 (22.3)	0.97	(0.90,1.05)				
Job insecurity	2,705	642 (23.7)	1.11	(1.02,1.20)	1.04	(0.96,1.12)	1.03	(0.95,1.11)
Number of distressing somatic symptoms in past week								
0	5,488	1,013 (18.5)	1		1		1	
1	1,991	523 (26.3)	1.33	(1.23,1.44)	1.28	(1.18,1.38)	1.19	(1.09,1.29)
2 +	1,628	535 (32.9)	1.54	(1.39,1.71)	1.43	(1.30,1.58)	1.25	(1.14,1.38)
Missing	54	11 (20.4)						
Mental health								
Good	3,623	731 (20.2)	1		1		1	
Intermediate	2,771	643 (23.2)	1.22	(1.09,1.36)	1.16	(1.04,1.29)	1.11	(1.00,1.24)
Poor	2,733	702 (25.7)	1.37	(1.24,1.51)	1.22	(1.12,1.34)	1.15	(1.05,1.26)
Missing	34	6 (17.6)						
Number of anatomical sites with pain in the past 12 months before baseline^1^								
0	3,188	466 (14.6)	1				1	
1	2,211	434 (19.6)	1.3	(1.14,1.48)			1.28	(1.16,1.43)
2	1,579	407 (25.8)	1.64	(1.43,1.88)			1.53	(1.36,1.72)
3	1,101	326 (29.6)	1.86	(1.61,2.15)			1.66	(1.44,1.92)
4 +	996	421 (42.3)	2.56	(2.23,2.93)			2.06	(1.78,2.39)
Not known	86	28 (32.6)						

^1^Knee pain and lower back pain were excluded

^2^Mutually adjusted estimates including all sex- and age-adjusted estimates in one model, excluding the effect of number of anatomical sites with pain the past 12 months before baseline

^3^Mutually adjusted estimates including all sex- and age-adjusted estimates in one model

## Discussion

In this study we showed that knee pain is common in working people even from younger ages, is frequently disabling, long-lasting and persistent). We also showed wide variation in the prevalence of reporting knee pain amongst people from different countries doing broadly similar types of work. We found that knee pain is rarely an isolated symptom and mostly co-occurs with pain elsewhere. The predictive factor with the strongest effect size for knee pain at follow-up was the number of painful anatomical sites at baseline. Other risk factors were older age, psychosocial aspects of work, somatisation, poor mental health and occupational physical demands including climbing up or down stairs and kneeling or squatting on an average working day which significantly increased the risk of knee pain at follow-up.

The estimated prevalence of knee pain amongst workers in this study aligns with rates from population surveys [1, 2, 9]. For example, Urwin and colleagues reported prevalence rates of pain lasting more than a week in the past month of 10%, 23% and 32% amongst community-dwelling women aged 16–44, 45–64 and 65–74 respectively, with corresponding figures of 15%, 21% and 27% amongst men [21]. As with other studies, rates were higher amongst women than men and increased with age. Knee pain causes limited mobility and impaired function, substantially impacting work. According to a UK study, lower limb osteoarthritis was associated with a 2.6-fold increased risk of health-related job loss over two years of follow-up amongst women aged 50–64 years at baseline [22]. Likewise, researchers in Portugal reported 2.25-increased risk of early exit from work amongst people aged 50–64 years caused by knee OA and found an increased risk associated with longstanding pain and/or pain interference from OA [10]. Similar results have been reported elsewhere [23–26]. It appears that the pain and its functional impact cause the majority of work disability, rather than OA itself [10]. This is crucial as demographic changes require people to work to older ages, making knee pain increasingly impactful on productivity and work ability. Also, since women are more significantly affected by knee pain and knee OA, and more women are in the workforce in their 50 s, 60 s and 70 s than in previous generations [27], the burden of knee pain is set to increase.

Regional pain and multisite pain are linked with poor mental health: anxiety and depression predict incident low back [28] and neck pain [29]. Amongst people with osteoarthritis, poor mental health is strongly associated with knee pain [15]. A systematic review confirmed a relationship between depression and knee pain in adults [30]. Although less studied, somatisation has been shown to increase the risk of knee pain persistence over 18-month follow-up [31]. Moreover, amongst people with knee pain consulting in primary care, somatic symptoms predicted poorer function 12 months later [32]. The findings on psychosocial work factors and knee pain are novel, although these factors are known to affect the risk of regional musculoskeletal pain at others sites like the low back and upper limb [33].

Occupational physical demands have been previously linked with knee OA [13]. In particular, physical loading, heavy lifting and kneeling/squatting appear to be risk factors. Climbing stairs has also been implicated in some studies, although a systematic review found the evidence “limited” for this risk factor [34]. The current study found a small, but significant, effect for reporting climbing stairs > 30 times/day (PRR: 1.18) and for kneeling/squatting > 1 h/day (PRR: 1.13) but not for lifting weights > 25 kg. Whilst these effects attained statistical significance, they are small and suggest that, although occupational physical demands can play a role in knee OA, especially at younger ages, this is not the most likely explanation for most of the reported knee pain in this study. The study lacked radiographic investigation to be certain of what proportion OA has caused. Another explanation for the associations might be that those frequently climbing stairs or kneeling/squatting might be more aware of their knee pain than sedentary individuals.

Patients with knee OA also have pain at other joint sites [35]. One explanation is that chronic knee pain caused by osteoarthritis might alter gait patterns, leading to biomechanical changes, increased stresses and more damage to other lower extremity joints [36]. Indeed, patients with bilateral medial compartment knee OA have been shown to shift body weight away from the painful knee to reduce loading, potentially causing damage to other lower extremity joints such as the ankle, hip, and contralateral knee [37]. However, it is more difficult to understand the relationship between pain at the other anatomical sites including neck, shoulder, wrist/hand and low back pain. Another possibility is polyarticular OA where familial patterns affect the symmetrical joint distribution. However, Felson and colleagues [35] showed that knee pain patients were at increased risk of pain in various joints, without a predilection for lower limb sites affected by altered gait or classic OA sites. Alternatively, knee pain, like other regional pain syndromes (low back pain, non-specific upper limb pain), is better explained by the phenomenon of central sensitisation, which involves amplification of pain signals within the central nervous system [38]. A systematic review showed that up to 56% of people with knee OA may experience central sensitisation [39]. Certainly, the risk factors for conditions associated with central sensitisation align well with those identified here, namely poor mental health, poor self-rated health and somatisation. This is particularly important for the treatment of patients with knee pain as approaches based on treatment of structural damage are likely to lead to an increased risk of poorer outcomes, chronic pain and treatment failure.

As with low back and neck/shoulder pain [40, 41], we found wide variation in knee pain prevalence by country, even amongst workers doing similar types of work (e.g. nurses). Previously, the number of painful anatomical sites at baseline was also the factor which best explained the risk of disabling low back and wrist/hand pain at follow-up within the CUPID study [18]. These country-specific variations have not been explained by availability of sick pay or workers’ compensation in different countries [18]. The consistency of these findings for pain at different anatomical sites continues to support the hypothesis that occupational physical demands are not the sole determinant of musculoskeletal pain and that therefore, prevention strategies focussed mainly on alleviating these demands will not be sufficient on their own to reduce the burden. That so few workers with knee pain only report knee pain, and that such wide variation is seen in rates of knee pain amongst workers doing similar jobs suggest that other determinants of disabling musculoskeletal pain exist. Our hypothesis is that psychological, psychosocial and cultural factors also play an important role in pain and disability, particularly pain in more than one site. These factors must be addressed if progress is to be made in reducing the substantial work disability caused by musculoskeletal disorders. For pain at other specific sites, the wide variation between occupational groups and countries correlated with variation in the prevalence of pain at other sites. For example, higher prevalence of low back pain seemed to be driven largely by higher propensity to report musculoskeletal pain in general. It is likely that the same is true for knee pain [18].

## Strengths and limitations

Our study’s strengths include a large, culturally diverse sample from 18 countries and a longitudinal design with a high follow-up response rate. Information was collected on numerous potential risk factors for knee pain using standardised questions, many derived from established instruments [20].

There are several limitations in this study. Firstly, the study sample comprised individuals aged 20–59 years at baseline from three occupational categories. However, those occupations are likely to be broadly representative of the wider adult population with regard to their experience of knee and other musculoskeletal pain, and the associations with related risk factors. Selective loss to follow-up due to work disability associated with pain (i.e. healthy worker effect) may have resulted in underestimated persistence and incidence rates of knee pain. Despite these, it is unlikely that these would have led to a systematic bias in the associations shown within these internal comparisons. Additionally, we were unable to differentiate between short and long duration of exposure to specific occupational activities, potentially diluting stronger effects of a long duration of exposure if there were large numbers with a short duration. Further study of the effects of duration/dose would require different study methods e.g. biomechanical studies using objective measurement techniques. Moreover, occupational activities exposure was reported approximately 14 months before reporting of the outcome measure, potentially mossing job changes during this period. However, sensitivity analyses restricting the study sample to those who remained in the same job over follow-up (95% of participants) showed that associations remained the same (data not shown). Lastly, we do not have information about participants’ BMI and history of prior knee injury. Previous research has shown that both are important risk factors of knee pain [42, 43]. Nevertheless, there is unlikely to be a systematic relationship between either of these factors and the risk factors explored here, like psychosocial aspects of work, occupational activities, and number of pain sites (outside back and knee pain) in the past 12 months.

## Conclusions

This study adds to the evidence that knee pain is common and disabling amongst workers. As its prevalence increases age and can be work-disabling, more attention on prevention and early intervention for knee pain are indicated. In particular however, our results point to the importance of a biopsychosocial approach to management of knee pain akin to that recommended for low back pain and non-specific arm pain as approaches focussed on structural damage to the knee alone are unlikely to be universally effective.

## Supplementary Information

Supplementary Material 1. Supplementary Tables A1 and A2.

## Data Availability

The dataset analysed for the current study is available from the corresponding author on reasonable request.

## Abbreviations

BMI: Body Mass Index
CI: Confidence Intervals
CUPID: Cultural and Psychosocial Influences on Disability
PRR: Prevalence Rate Ratios
