# Supplementary material for: Longitudinal study of knee pain amongst workers in the Cultural and Psychosocial Influences on Disability (CUPID) study
Source: BMC Musculoskelet Disord. 2025 Nov 5;26:1026. doi: 10.1186/s12891-025-09237-z (PMC12590909; doi:10.1186/s12891-025-09237-z)
Supplement: Supplementary file 1 — Supplementary Material 1. Supplementary Tables A1 and A2. [file 12891_2025_9237_MOESM1_ESM.docx]

**Supplementary Table A1.** Response rates at follow-up by occupational group

| **Country/Occupational Group** |  | Number with baseline data | Number with follow-up data | Response rates (%) |
| --- | --- | --- | --- | --- |
| **Brazil** |  |  |  |  |
| Nurses |  | 41 | 135 | 76.7 % |
| Office workers |  | 42 | 226 | 84.3 % |
| Manual workers |  | 49 | 42 | 46.2 % |
| **Ecuador** |  |  |  |  |
| Nurses |  | 16 | 201 | 92.6 |
| Office workers |  | 22 | 221 | 90.9 |
| Manual workers |  | 14 | 212 | 93.8 |
| **Colombia** |  |  |  |  |
| Office workers |  | 17 | 75 | 81.5 |
| **Costa Rica** |  |  |  |  |
| Nurses |  | 21 | 196 | 90.3 |
| Office workers |  | 20 | 202 | 91.0 |
| **Nicaragua** |  |  |  |  |
| Nurses |  | 15 | 266 | 94.7 |
| Office workers |  | 31 | 254 | 89.1 |
| Manual workers |  | 38 | 158 | 80.6 |
| **UK** |  |  |  |  |
| Nurses |  | 117 | 135 | 53.6 |
| Office workers |  | 150 | 225 | 60.0 |
| Manual workers |  | 223 | 145 | 39.4 |
| **Spain** |  |  |  |  |
| Nurses |  | 89 | 578 | 86.7 |
| Office workers |  | 46 | 392 | 89.5 |
| **Italy** |  |  |  |  |
| Nurses |  | 152 | 374 | 71.1 |
| Manual workers |  | 38 | 90 | 70.3 % |
| **Greece** |  |  |  |  |
| Nurses |  | 8 | 216 | 96.4 % |
| Office workers |  | 6 | 193 | 97.0 % |
| Manual workers |  | 12 | 127 | 91.4 % |
| **Estonia** |  |  |  |  |
| Nurses |  | 225 | 143 | 38.9 % |
| Office workers |  | 94 | 108 | 53.5 % |
| **Lebanon** |  |  |  |  |
| Nurses |  | 71 | 113 | 61.4 |
| Office workers |  | 40 | 132 | 76.7 |
| Manual workers |  | 34 | 103 | 75.2 |
| **Iran** |  |  |  |  |
| Nurses |  | 27 | 219 | 89.0 |
| Office workers |  | 16 | 166 | 91.2 |
| **Pakistan** |  |  |  |  |
| Nurses |  | 16 | 165 | 91.2 |
| Office workers |  | 15 | 161 | 91.5 |
| Manual workers |  | 13 | 198 | 93.8 |
| **Sri Lanka** |  |  |  |  |
| Nurses |  | 20 | 215 | 91.5 % |
| Office workers |  | 10 | 142 | 93.4 % |
| Manual workers^1^ |  | 47 | 197 | 80.7 % |
| Manual workers^1^ |  | 6 | 145 | 96.0 % |
| **Japan** |  |  |  |  |
| Nurses |  | 244 | 341 | 58.3 % |
| Office workers |  | 61 | 247 | 80.2 % |
| Manual workers^1^ |  | 334 | 656 | 66.3 % |
| Manual workers^1^ |  | 47 | 305 | 86.6 % |
| **South Africa** |  |  |  |  |
| Nurses |  | 56 | 191 | 77.3 % |
| **Australia** |  |  |  |  |
| Nurses |  | 69 | 180 | 72.3 % |
| **New Zealand** |  |  |  |  |
| Nurses |  | 24 | 152 | 86.4 % |
| Office workers |  | 9 | 135 | 93.8 % |
| Manual workers |  | 29 | 84 | 74.3 % |

^1^ two separate groups of manual workers as per main CUPID methods paper [20]

**Supplementary Table A2**. Associations of disabling knee pain in the past month as reported at follow-up with baseline personal and occupation risk factors

| **Risk factor** | **All** | **Knee pain at follow-up** |  | **Sex- and age-adjusted estimates** | |  | **Mutually adjusted estimates** | |  | **Mutually adjusted estimates** | |
| --- | --- | --- | --- | --- | --- | --- | --- | --- | --- | --- | --- |
|  | **N** | **N (%)** |  | **PRR** | **(95%CI)** |  | **PRR^2^** | **(95%CI)** |  | **PRR^3^** | **(95%CI)** |
| **Sex** |  |  |  |  |  |  |  |  |  |  |  |
| Male | 2,931 | 371 (12.7) |  | 1 |  |  | 1 |  |  | 1 |  |
| Female | 5,708 | 1,189 (20.8) |  | 1.4 | (1.17,1.66) |  | 1.27 | (1.07,1.50) |  | 1.17 | (0.99,1.37) |
| **Age (years)** |  |  |  |  |  |  |  |  |  |  |  |
| 20-29 | 2,010 | 250 (12.4) |  | 1 |  |  | 1 |  |  | 1 |  |
| 30-39 | 2,769 | 426 (15.4) |  | 1.24 | (1.07,1.45) |  | 1.27 | (1.10,1.47) |  | 1.19 | (1.04,1.36) |
| 40-49 | 2,477 | 508 (20.5) |  | 1.59 | (1.35,1.86) |  | 1.64 | (1.41,1.90) |  | 1.47 | (1.28,1.70) |
| 50-59 | 1,383 | 376 (27.2) |  | 2.13 | (1.80,2.53) |  | 2.21 | (1.88,2.61) |  | 1.91 | (1.66,2.21) |
| **Smoking** |  |  |  |  |  |  |  |  |  |  |  |
| Never smoked | 5,577 | 1,111 (19.9) |  | 1 |  |  | 1 |  |  | 1 |  |
| Ex-smoker | 1,234 | 215 (17.4) |  | 1 | (0.88,1.15) |  | 0.99 | (0.86,1.14) |  | 0.96 | (0.84,1.10) |
| Current smoker | 1,805 | 228 (12.6) |  | 0.86 | (0.77,0.98) |  | 0.8 | (0.71,0.91) |  | 0.78 | (0.69,0.89) |
| Not known | 6 | 6 (26.1) |  |  |  |  |  |  |  |  |  |
| **Activity in average working day** |  |  |  |  |  |  |  |  |  |  |  |
| Lifting weights ≥25 kg | 3,089 | 570 (18.5) |  | 1.14 | (1.01,1.28) |  | 1.03 | (0.91,1.15) |  | 1.01 | (0.90,1.13) |
| Climbing up or down more than 30 flights of stairs | 1,953 | 437 (22.4) |  | 1.26 | (1.11,1.42) |  | 1.18 | (1.06,1.32) |  | 1.18 | (1.05,1.33) |
| Kneeling or squatting for >1hr | 2,325 | 510 (21.9) |  | 1.3 | (1.19,1.42) |  | 1.21 | (1.11,1.31) |  | 1.13 | (1.04,1.24) |
| **Psychosocial aspects of work** |  |  |  |  |  |  |  |  |  |  |  |
| Work for >50 hours per week | 1,961 | 282 (14.4) |  | 1.06 | (0.95,1.17) |  |  |  |  |  |  |
| Time pressure at work | 6,460 | 1,244 (19.3) |  | 1.39 | (1.24,1.55) |  | 1.29 | (1.17,1.43) |  | 1.25 | (1.12,1.39) |
| Incentives at work | 2,408 | 458 (19.0) |  | 1.12 | (0.98,1.27) |  |  |  |  |  |  |
| Lack of support at work | 2,197 | 465 (21.2) |  | 1.15 | (1.04,1.28) |  | 1.12 | (1.01,1.25) |  | 1.09 | (0.98,1.21) |
| Job dissatisfaction | 1,674 | 274 (16.4) |  | 1.09 | (0.95,1.25) |  |  |  |  |  |  |
| Lack of job control | 1,733 | 303 (17.5) |  | 0.95 | (0.85,1.05) |  |  |  |  |  |  |
| Job insecurity | 2,539 | 476 (18.7) |  | 1.12 | (1.02,1.22) |  | 1.03 | (0.94,1.13) |  | 1.02 | (0.94,1.11) |
| **Number of distressing somatic symptoms in past week** |  |  |  |  |  |  |  |  |  |  |  |
| 0 | 5,165 | 690 (13.4) |  | 1 |  |  | 1 |  |  | 1 |  |
| 1 | 1,884 | 416 (22.1) |  | 1.47 | (1.32,1.63) |  | 1.42 | (1.28,1.57) |  | 1.29 | (1.17,1.43) |
| 2+ | 1,536 | 443 (28.8) |  | 1.74 | (1.51,1.99) |  | 1.6 | (1.40,1.84) |  | 1.36 | (1.19,1.54) |
| Missing | 54 | 11 (20.4) |  |  |  |  |  |  |  |  |  |
| **Mental health** |  |  |  |  |  |  |  |  |  |  |  |
| Good | 3,454 | 562 (16.3) |  | 1 |  |  | 1 |  |  | 1 |  |
| Intermediate | 2,595 | 467 (18.0) |  | 1.21 | (1.07,1.37) |  | 1.13 | (1.01,1.26) |  | 1.08 | (0.96,1.21) |
| Poor | 2,557 | 526 (20.6) |  | 1.41 | (1.25,1.59) |  | 1.22 | (1.09,1.37) |  | 1.12 | (1.00,1.26) |
| Missing | 33 | 5 (15.2) |  |  |  |  |  |  |  |  |  |
| **Number of anatomical sites with pain in the past 12 months before baseline^1^** |  |  |  |  |  |  |  |  |  |  |  |
| 0 | 3,040 | 318 (10.5) |  | 1 |  |  |  |  |  | 1 |  |
| 1 | 2,074 | 297 (14.3) |  | 1.36 | (1.17,1.58) |  |  |  |  | 1.3 | (1.12,1.51) |
| 2 | 1,473 | 301 (20.4) |  | 1.76 | (1.52,2.02) |  |  |  |  | 1.62 | (1.42,1.86) |
| 3 | 1,038 | 263 (25.3) |  | 2.11 | (1.76,2.54) |  |  |  |  | 1.89 | (1.58,2.27) |
| 4+ | 936 | 361 (38.6) |  | 2.88 | (2.35,3.52) |  |  |  |  | 2.4 | (1.99,2.91) |
| Not known | 78 | 20 (25.6) |  |  |  |  |  |  |  |  |  |

^1^Knee pain and lower back pain were excluded ^2^Mutually adjusted estimates including all sex- and age-adjusted estimates in one model, excluding the effect of number of anatomical sites with pain the past 12 months before baseline ^3^Mutually adjusted estimates including all sex- and age-adjusted estimates in one model
